# P-2253. Epidemiology and Outcomes of Gram-Negative Bacteremia in a Diverse Immunocompromised Population

**DOI:** 10.1093/ofid/ofae631.2406

**Published:** 2025-01-29

**Authors:** Anahita Mostaghim, Nehal G Hashem, Sabina Pathan, Alison Han

**Affiliations:** Atrium Health, Charlotte, North Carolina; NIH, Bethesda, Maryland; National Institute of Allergy and Infectious Disease, Bethesda, Maryland; National Institutes of Health, Bethesda, Maryland

## Abstract

**Background:**

While gram-negative bloodstream infections (GN-BSI) carry significant risk of mortality and are of particular concern in neutropenic patients, relatively little is known about epidemiology and outcomes in contemporary immunocompromised populations. This study aims to assess the epidemiology of GN-BSI admitted to the National Institutes of Health Clinical Center (NIH CC).

**Figure:**
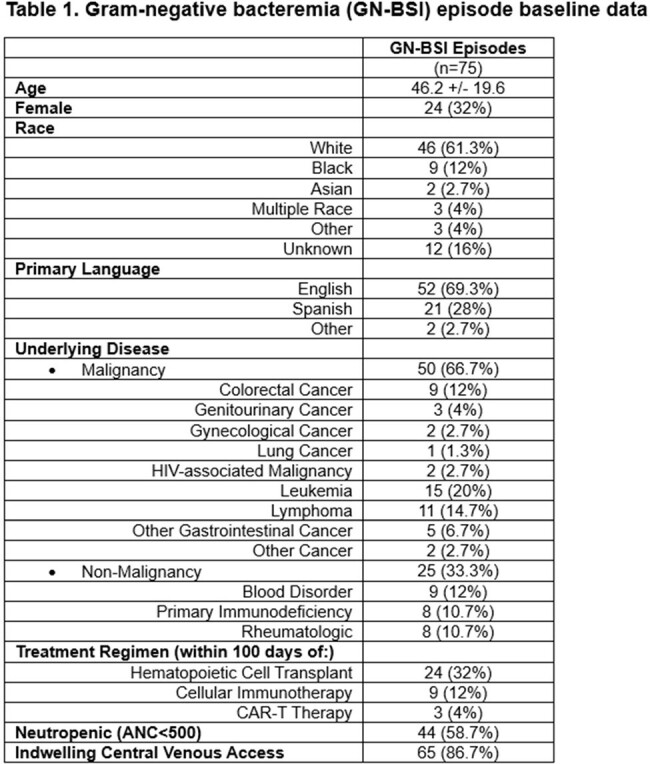

**Methods:**

Episodes of GN-BSI were identified through positive blood culture results from January 1 to December 31, 2023. Episodes not treated at the NIHCC were excluded. We performed chart review for baseline demographics, laboratory results, infectious disease consultation results, and clinical outcomes.

**Results:**

Seventy-five episodes of GN-BSI admitted to the NIHCC were identified. All patients were immunocompromised by disease or treatment (Table 1). Forty-four (58.7%) were neutropenic at index culture with median duration of neutropenia 9 days after index culture (IQR 7-17.5) in survivors. Sixty-five (86.7%) had central venous access device (CVAD) and 16 (24.6%) were removed during the episode. Translocation (n=38, 50.8%) was the most frequently reported source followed by gastrointestinal (n=10, 13.3%) and pulmonary (n=9, 12%). *Escherichia coli* (n=25), *Klebsiella pneumonia* (n=17), and *Pseudomonas aeruginosa* (n=15) were the most frequently identified isolates. Twenty-one (28%) had polymicrobial bacteremia with 7 (33.3%) also containing a gram-positive isolate. Bacteremia duration was a single day in 64 (88.9%) episodes. Twenty-five (32.9%) had ICU admission and 7 (9.2%) experienced 30-day mortality. Neutropenic episodes had similar 30-day mortality (6.8% v 12.9%, OR 0.50, p=0.62) and ICU admission rate (OR 1.09, 0.37-3.28, p >0.99). Polymicrobial episodes had similar 30-day mortality (4.8% v 11.1%, p=0.45). Those with CVAD removal had lower rate of ICU admission (12.5% vs 37.3%, OR 0.16, p=0.03) and lower 30-day mortality (0% v 11.9%, p=0.22) though not statistically significant.

**Conclusion:**

*Enterobacteriaceae* caused most of GN-BSI at an institution with a diverse immunocompromised population. CVAD removal remains important in the treatment of GN-BSI. Neutropenia is not associated with worse outcomes.

**Disclosures:**

All Authors: No reported disclosures

